# Withdrawal: Astragaloside IV combined with quercetin attenuates silica-induced pulmonary fibrosis by promoting autophagy and suppressing pyroptosis

**DOI:** 10.1371/journal.pone.0333459

**Published:** 2025-10-01

**Authors:** The PLOS One Editors

This article [1] was published in error by PLOS after the authors requested withdrawal. Therefore, *PLOS One* has withdrawn this article [1] and removed the article contents from the journal website. The publisher apologizes for the error.
